# Muskelverletzungen: Stellenwert der hochauflösenden dynamischen Sonographie in der Diagnostik, Therapie und im Monitoring

**DOI:** 10.1007/s00132-024-04505-7

**Published:** 2024-05-13

**Authors:** Jörg Dünkel, Thomas-Oliver Scheider, Giorgio Tamborrini

**Affiliations:** 1 Sportklinik Bern, Bümplizstrasse 83, 3018 Bern, Schweiz; 2 Kniechirurgie Bern, Bern, Schweiz; 3 UZR – Schweizer Ultraschallzentrum und Institut für Rheumatologie, Basel, Schweiz; 4 Klinik für Rheumatologie, Universitätsspital Basel, Basel, Schweiz

**Keywords:** Verlaufskontrollen, Hamstringsehnen, MRT, Sportverletzung, Ultraschallbildgebung, Follow-up care, Hamstring tendons, MRI, Sports injuries, Ultrasound imaging

## Abstract

**Hintergrund:**

Im Fußballsport sind Muskelverletzungen ein häufiges Verletzungsmuster. Die Bildgebung ist ein zentrales Element zur Diagnosestellung. Hierfür werden hauptsächlich die MRT und der Ultraschall eingesetzt. Beide Verfahren haben Vor- und Nachteile, welche abgewogen werden sollten.

**Neue Ultraschalltechniken:**

Die Rolle der MRT als Goldstandard wird zunehmend durch neue Techniken des hochauflösenden Ultraschalls abgelöst und nicht immer ist eine MRT-Bildgebung sinnvoll. Auch während der Reha-Phase empfehlen sich regelmäßige Ultraschallbildgebungen, um Komplikationen frühzeitig zu erkennen. Hierbei kann der Heilungsverlauf monitorisiert werden, und es besteht die Möglichkeiten für ultraschallnavigierte Interventionen, wie Hämatompunktionen und gezielte Infiltrationsbehandlungen.

**Vorteile und Nachteile:**

Ein Nachteil der Ultraschalldiagnostik ist jedoch die Nutzerabhängigkeit. Bei erfahrenen Anwendern wird diese durch die zahlreichen Vorteile des modernen Ultraschalls ausgeglichen, womit er der MRT in vielen Bereichen – speziell auch mit der Möglichkeit eines dynamischen Ultraschalls – überlegen ist. Dennoch bleibt die MRT bei bestimmten Fragestellungen eine sinnvolle und notwendige Untersuchungsmethode.

## Abstract (en)

**Background:**

Muscle injuries are common in football. Imaging diagnostics have a major role in establishing a diagnosis. The main diagnostic procedures are MRI and ultrasound. Both diagnostics have advantages and disadvantages, which should be balanced against each other.

**New ultrasonic techniques:**

The role of MRI as the gold standard is increasingly being replaced by high-resolution ultrasound techniques, and MRI imaging is not always useful. To detect complications in the early stages it is advised to perform regular ultrasound-imaging check-ups. The healing process can be monitored, and it offers additional options for ultrasound-guided interventions such as hematoma punctures and targeted infiltrations.

**Advantages and disadvantages:**

However, ultrasound imaging is highly user dependent. Experienced operators can eliminate this disadvantage, which makes ultrasound a superior imaging system in many areas, especially for dynamic examinations. Nevertheless, MRI imaging remains a necessary imaging method in certain areas.

Die Diagnose und Therapie von Muskelverletzungen im Fußballsport zählen zu den täglichen Herausforderungen der medizinischen Abteilung eines Profivereins. Zur Diagnosesicherung ist heute die Bildgebung das zentrale Element für die Abschätzung der Ausfallzeit und der weiteren Therapieplanung. Durch technische Innovationen hat sich in den vergangenen Jahren die Ultraschallbildgebung soweit entwickelt, dass die MRT auch im Spitzensport nicht mehr als alleiniger Goldstandard gesehen werden kann. Beide Verfahren haben Vor- und Nachteile, welche bei der Anwendung bedacht werden müssen.

Muskelverletzungen im Fußballsport zählen zu den regelmäßigen Verletzungen und führen zu langen Ausfallzeiten [7]. Gerade bei Teams mit Dreifachbelastungen (Meisterschaft, Pokal- und internationaler Wettbewerb) treten aufgrund der hohen Spielbelastung z. B. in „Englischen Wochen“ häufiger Muskelverletzungen auf, als im normalen wöchentlichen Spielbetrieb [22]. Als einer der Teamärzte des Berner Sport Club Young Boys (BSC YB) ist Dr. med. Jörg Dünkel hauptverantwortlicher Arzt für die Betreuung von Muskelverletzungen der ersten Mannschaft sowie auch der Nachwuchsspieler*innen. Der BSC YB ist ein Spitzenteam der ersten Schweizer Liga (Swiss Super League), gewann in den vergangenen 6 Jahren fünfmal die Meisterschaft, war in diesem Zeitraum zweimal Schweizer Cup Sieger und nahm bis auf die Saison 22/23 immer an internationalen Gruppenphasen Teil – zweimal davon in der Champions League und dreimal in der Europa League.

Eine genaue und schnelle Diagnose bei Muskelverletzungen ist entscheidend für die Reha-Planung und eine schnelle und sichere Rückkehr zum normalen Spielbetrieb. Neben der Anamnese, Analyse von Bildmaterial des Unfallereignisses (wenn vorhanden) und der klinischen Untersuchung ist die Bildgebung das zentrale Element für die Diagnosestellung. Auch zur Verlaufskontrolle wird diese neben dem klinischen Verlauf immer häufiger zur Überwachung eingesetzt. Hierbei kommen hauptsächlich die MRT und der hochauflösende Ultraschall zum Einsatz. Sowohl die MRT als auch der Ultraschall (US) haben sich in den vergangenen Jahren technisch weiterentwickelt, sodass die Bildqualität deutlich verbessert werden konnte. So können mittels 3 T-MRT das Ödem besser von der strukturellen Läsion abgegrenzt und mittels hochauflösenden Ultraschalles auch kleine strukturelle Pathologien dargestellt werden. Beide bildgebenden Verfahren haben Vor- und Nachteile (Tab. 1).

**Table 1:** 

	Ultraschall	MRT
*Vorteile*	Schnelle Verfügbarkeit (auch portable Geräte)	Hohe Sensitivität (insb. Flüssigkeit/kleine Hämatome)
	Sehr hohe Auflösung (0,1 mm)	Auflösung bis 1 mm* (optimal anguliert koronar und sagittal – axial 3–5 mm)
	Kostengünstig	Erfassung von Begleitverletzungen in angrenzenden oder entfernten Regionen (wenn relevant)
	Dynamische Untersuchungen	Tiefe Muskulatur
	Unkompliziert für Patienten	Differenzierung von Rezidivverletzungen in Narbengewebsbereichen
	Serielle Untersuchungen	
	Frühes Erfassen von Komplikationen und gleichzeitiger Therapie	
	Ultraschallnavigierte Interventionen	
	Zusätzliche Optionen wie Doppler, Mikrovaskularisation, Elastographie, Panoramaaufnahmen, 3‑D-Darstellung	
	Echopalpation	
	Vergleich zur kontralateralen Seite	
	Keine Kontraindikationen (Herzschrittmacher)	
*Nachteile*	Nutzerabhängig	Untersucherabhängig
	Reduzierte Sensitivität bei niedriggradigen Verletzungen (Ödem)	Risiko der Überschätzung von Verletzungen (Ödem)
	Limitierte Darstellung von tiefen Strukturen/Muskeln	Kostenintensiv
	Darstellung von sehr kleinen Sehnenverletzungen (z. B. Längsrupturen der proximalen Hamstringsehnen) begrenzt	Eingeschränkte Verfügbarkeit
	M. soleus ist (zumindest initial) nicht sicher beurteilbar	Serielle Untersuchungen nicht sinnvoll (Kosten, Aufwand)
	Unterscheidung von Rezidivverletzungen im Narbengewebe benötigen sehr große Erfahrung und Training	Zeigt Veränderungen über Monate nach der Verletzung
		Komplikationen wie z. B. Myositis ossificans können erst im Verlauf diagnostiziert werden, fehlende Früherkennung

*Für 3 T-Geräte – 1,5 T-Geräte zeigen schlechtere Auflösungen > 5 mm axial und > 3 mm koronar und sagittal

Die Ultraschallbildgebung bleibt trotz aller technischen Innovationen ein nutzerabhängiges Verfahren. Im Gegensatz zur MRT werden keine standardisierten Schnittbilder erzeugt, sondern das Bildmaterial wird von vielen Variablen wie Frequenz, Schallkopf und Lagewinkel des Schallkopfs, um nur einzige zu nennen, beeinflusst. Diese können aber auch als Vorteil genutzt werden, welcher bei richtiger Anwendung eine Vielzahl von diagnostischen Möglichkeiten eröffnet. Eine statische MRT-Bildgebung ist diesbezüglich limitiert. Fundierte anatomische Kenntnisse der Muskel- und Sehnenstrukturen sind die Basis für jeden Untersucher. Erst durch jahrelanges Training sowie die Verwendung eines leistungsstarken modernen Ultraschallgeräts können präzise auch winzige Verletzungen und Veränderungen diagnostiziert werden, welche bisher hauptsächlich eine Domäne der MRT-Bildgebung waren.

Der Weg zur Diagnose führt im Profifußball immer über eine Bildgebung

Das Wichtigste bei einer Verletzung ist die Diagnosestellung. Hierfür ist in der heutigen Zeit im Profisport nach der klinischen Untersuchung und Stellung einer Verdachtsdiagnose immer eine Bildgebung notwendig. Das Risiko einer übersehenen Verletzung ist aufgrund der enormen finanziellen Risiken für Spieler und Vereine zu groß, um mit klinischen Verdachtsdiagnosen die weitere Belastung zu steuern. Auch der mentale Aspekt des Spielers, der in Sorge um eine allfällige Verletzung nicht mehr den vollen Einsatz leistet, ist hierbei nicht zu unterschätzen. Durch enge Anbindung an ein spezialisiertes medizinisches Team ist der Zugang zur MRT- und Ultraschall-Bildgebungen in kürzester Zeit verfügbar. Teilweise sind diese Geräte im Stadion oder deren unmittelbarer Umgebung stationiert. Zusätzlich bieten kleine mobile Ultraschallgeräte im Taschenformat schon heute eine ordentliche Bildqualität, welche direkt in der Kabine zum Einsatz kommen können. Mobile Handgeräte sind aufgrund ihrer eingeschränkten Bildqualität allerdings auf erfahrene Anwender angewiesen. Diese bieten im Gegenzug jedoch eine gute Ergänzung zur klinischen Untersuchung.

Aufgrund solcher Möglichkeiten besteht eine entsprechende Erwartung der Vereine auf eine rasche Diagnose. In der heutigen digitalisierten und schnelllebigen Zeit mit entsprechender sofortiger Verfügbarkeit von Informationen steht das medizinische Team eines Vereins zunehmend unter Druck, umgehend eine Diagnose zu liefern. Der Zeitpunkt für eine Bildgebung wird in der Literatur jedoch zwischen 2 und 48 h für eine MRT-Bildgebung (optimal nach 48 h) und 72 h nach der Verletzung für eine Ultraschall-Bildgebung angegeben [1, 3, 8, 14, 19, 35]. Eine zu frühe Diagnostik kann gerade bei niedriggradigen Verletzungen ein falsch negatives Ergebnis liefern [21]. Unabhängig hiervon wird ein Profiverein kaum 2–3 Tage bis zur Diagnostik warten können. Bei einem straffen Spielplan mit nationalen und internationalen Spielen alle 2–3 Tage ist dies in der Praxis nicht realistisch. Auf Basis dieser Tatsachen schlagen wir ein angepasstes Diagnostikschema vor (Abb. 1). Hierbei wird den Realverhältnissen im Profifußball bei der Diagnostik in der hyperakuten Phase (unter 2 h nach Verletzung) und deren Verifizierung im weiteren Verlauf Rechnung getragen.

**Figure 1:**
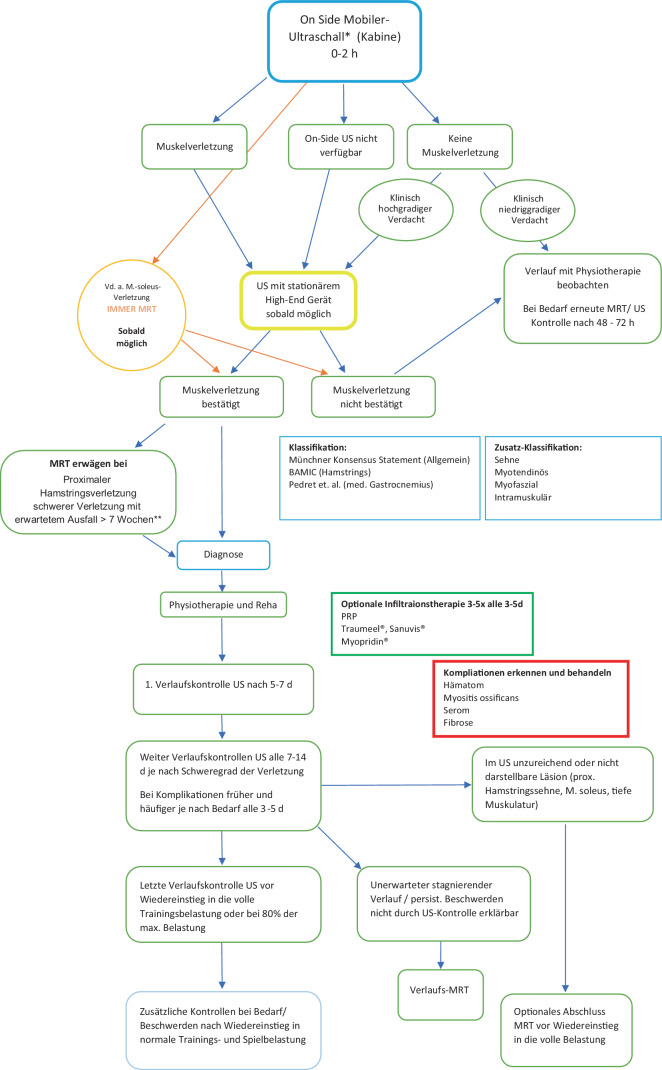

## MRT oder Ultraschall – was ist die richtige Diagnostik?

Diese Frage ist maßgeblich von den vorhandenen Ressourcen abhängig. Steht kein ausgewiesener Experte für die Ultraschalluntersuchung zur Verfügung, verfällt diese Option und eine MRT-Bildgebung ist unumgänglich. Bei der Möglichkeit, aus beiden Optionen zu wählen, stellt sich nun die Frage, welches Untersuchungsverfahren zum Einsatz kommt. Ein erfahrener Untersucher kann mittels Ultraschall einen Großteil der Diagnosen sicher und zuverlässig stellen. Dennoch gibt es Bereiche, wo eine Ultraschalluntersuchung an ihre Grenzen kommt. Dies sind v. a. Verletzungen von tiefen Gewebestrukturen (z. B. tiefe Hüftmuskulatur wie M. piriformis, M. iliopsoas [34, 35]), proximale Ursprungsverletzungen der Hamstringsehnen und der M. soleus. Bei tiefen Muskelpartien sinkt die Auflösung und teilweise sind diese aufgrund von ossären Strukturen nicht vollständig einsehbar. Im Bereich der proximalen Hamstringsehnen können eventuell kleinere Längsrupturen übersehen werden [19] und der M. soleus ist aufgrund seiner Anatomie (komplexe Bindegewebsstrukturen mit drei intramuskulären Aponeurosen/Sehen) sonographisch – zumindest in der Akutphase – nicht sicher beurteilbar [13]. Während das Hauptproblem der Ultraschallbildgebung die Nutzerabhängigkeit ist, so liefert die MRT aufgrund ihrer hohen Sensitivität teilweise Befunde, welche einer besonderen Interpretation bedürfen. So können „überstrahlende“ Ödeme und Hämatome zu einer Überinterpretation der Verletzung führen, was dann möglicherweise eine nicht optimale Reha-Planung nach sich zieht.

**Figure 2:**
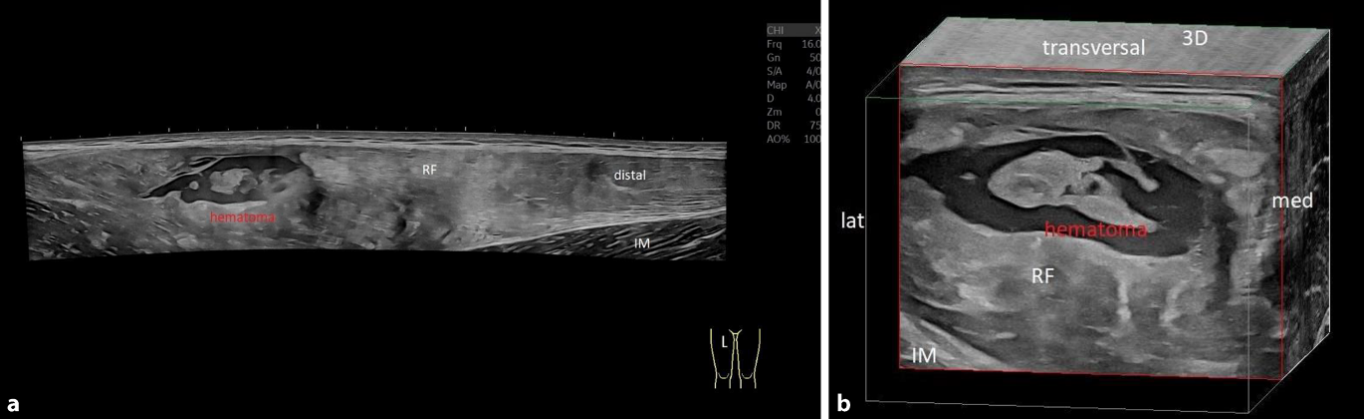

Moderne hochauflösende Ultraschallgeräte mit wassergekühlten Matrixschallköpfen und variablen Frequenzbereichen (z. B. GE ML 4–20 MHz Sonde, General Electric, Boston, MA, USA) sind in der Bildqualität und auch der Einsehbarkeit von tiefergelegenen Strukturen der MRT-Bildgebung heutzutage sicher ebenbürtig. Bei oberflächlichen und gut zugänglichen Regionen ist die Ultraschalluntersuchung aufgrund der höheren Auflösung der MRT teilweise überlegen. Zusätzlich ist es möglich, mittels Panoramaaufnahmen großflächige Bereiche in einem zusammenhängenden Bild darzustellen und 3‑D-Rekonstruktionen erzeugen hochauflösende Schnittbilder (Abb. 2a, b: Muskelbündelriss mit 3‑D-Rekonstruktion). Des Weiteren kommen dynamische Untersuchungen, der Doppler-Ultraschall, die Power-Doppler-Sonographie und die Elastographie zum Einsatz. Bis auf wenige erwähnte Ausnahmen, ist die Ultraschallbildgebung somit ein hervorragendes Instrument für die Darstellung von Muskelverletzungen und kann daher auch im Profisport als diagnostische Methode der Wahl zum Einsatz kommen.

## Beispiel eines Zufallsbefundes in der MRT

**Figure 3:**
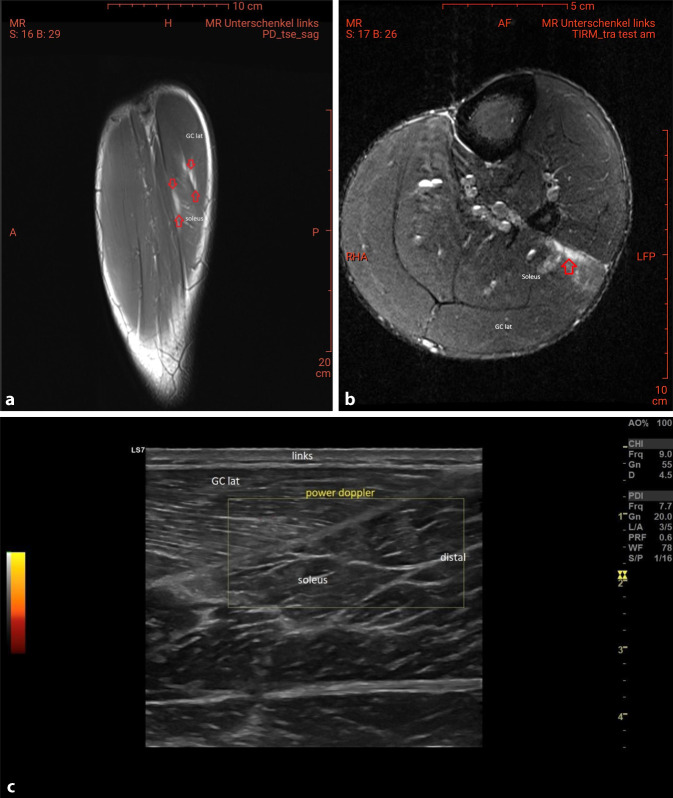

Manchmal ergeben sich in der MRT Befunde von fraglicher klinischer Relevanz, jedoch mit enormen Auswirkungen auf die Sport- und Spielfähigkeit einzelner Spieler. Teilweise liegen diese in Regionen, welche nur zufällig miterfasst wurden. In einem solchen Fall wurde einer unserer gesunden Spieler, welcher im vollen Trainings- und Spielbetrieb stand, für einen Transfer beim neuen Verein einer MRT der unteren Extremitäten unterzogen. Hierbei zeigte sich eine ödematöse Veränderung des M. soleus und des M. gastrocnemius (Abb. 3a–c). Der Spieler war zwar klinisch asymptomatisch und im Ultraschall konnte der Befund nicht korreliert werden, dennoch wurde er aufgrund des MRT-Befundes aus dem Spielbetrieb genommen und „saß“ seine restliche Zeit bis zum Saisonende und dem anstehenden Transfer auf der Tribüne ab.

## Einteilung und Klassifikation

Eine allgemeine und generelle Klassifikation von Muskel- und Sehnenverletzungen ist nicht vorhanden. So existieren acht verschiedene Klassifikationen auf Basis von Klinik und Bildgebungen, acht auf Basis von Bildgebungen und zwei weitere für klinische Untersuchungen [26]. Zusätzlich gibt es bereits Klassifikationen von einzelnen Muskeln und Muskelgruppen [20, 23]. Ein Hauptaspekt ist die Abschätzung der zu erwartenden Ausfallzeit, welche auf einzelne Bereiche und Muskelgruppen besser spezifiziert werden kann, als bei globalen Klassifikationen. Denn nach dieser richtet sich maßgeblich der Reha- und Aufbauplan.

Einige gängige Klassifikationen sind die Münchner Konsensus Klassifikation [17], die British Athletics muscle injury classification (BAMIC – speziell für Hamstringverletzungen) und die Klassifikation der medialen Gastroknemiusverletzungen nach Pedret et al. [20], welche im Gegensatz zu vielen anderen Klassifikationen auch die Ausfallzeit je nach Verletzungsausmaß definieren.

Zudem rückt in letzter Zeit immer mehr der Aspekt des Ausmaßes des verletzten Binde- und Stützgewebes zur Einteilung des Schweregrades einer Verletzung in den Vordergrund [31]. So definieren sich proximale Sehnenverletzungen – insbesondere der freien Sehnen (z. B. der Hamstrings) – als besonders schwerwiegend mit langen Ausfallzeiten, wohingegen Verletzungen ohne Beteiligung von Stützgewebe (z. B. reine intramuskuläre Verletzungen) als weniger schwerwiegend mit kürzen Ausfallzeiten gesehen werden.

## Verlaufskontrollen

**Figure 4:**
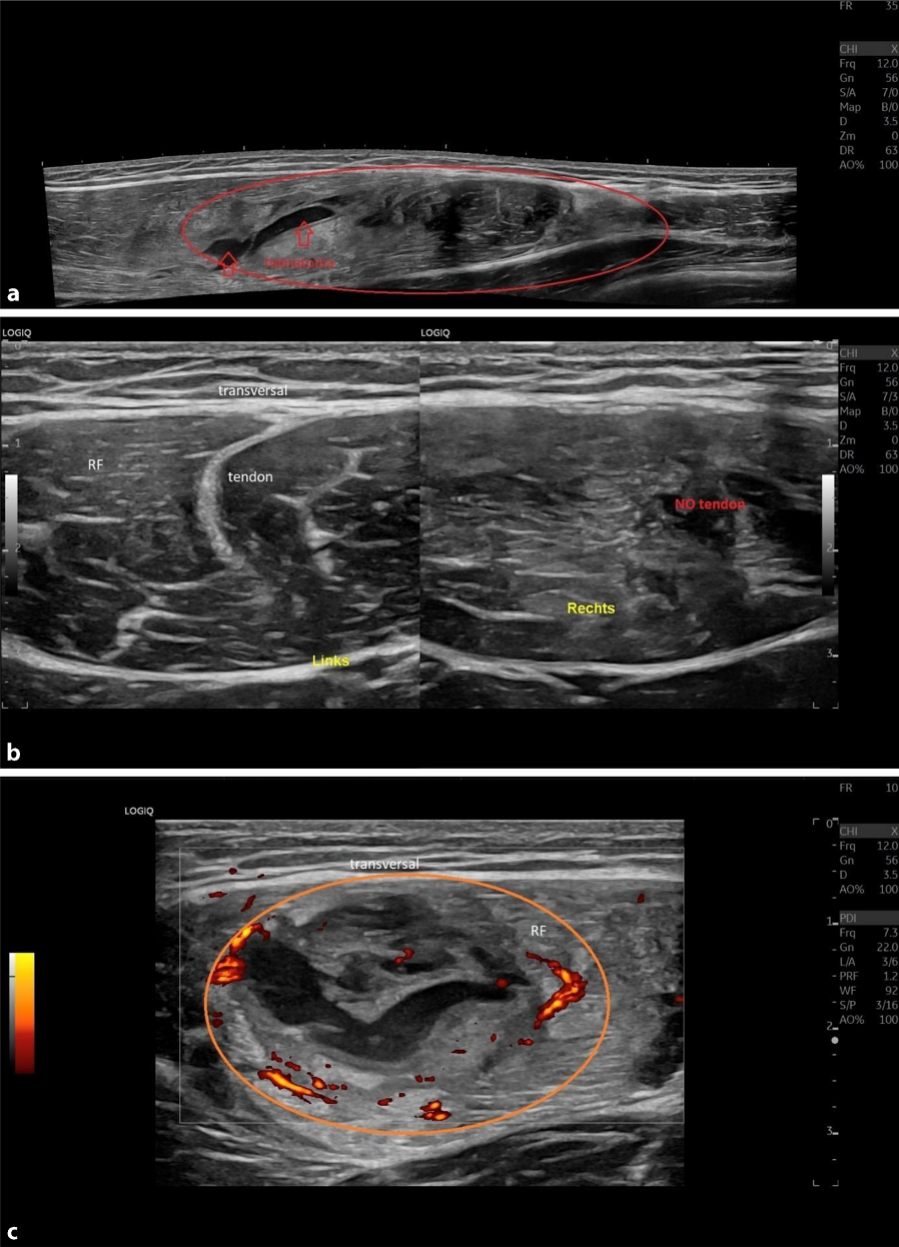

Ist die Diagnose einer Muskelverletzung gestellt, beginnt die Rehabilitation. Ein allgemeingültiges oder standardisiertes Reha-Konzept ist aufgrund der Heterogenität der Verletzungen nicht vorhanden und variiert von Verein zu Verein. Zur klinischen Verlaufskontrolle empfiehlt sich eine begleitende Bildgebung, wofür sich der Ultraschall besonders gut eignet. Der Heilungsverlauf kann monitorisiert und allfällige Komplikationen frühzeitig erkannt werden. Hierbei zeigen sich typische Veränderungen, wie Resorption von Flüssigkeiten bei Hämatomen und Ödemen sowie Bildung von Narbengewebe und schließlich Wiederherstellung von normalen Muskelgewebestrukturen [6, 35]. Zusätzlich können auch der Doppler-Ultraschall, der Power-Doppler, die Elastographie und eine dynamische Ultraschalluntersuchung Informationen über den Verlauf der Verletzung und allfällige Komplikationen liefern ([5]; Abb. 4a–c).

## Doppler-Ultraschall, z. B. Farb-Doppler (C-Doppler), Power-Doppler, B-Flow, MVI

Direkt nach der Verletzung besteht eine Hyperämie in der Doppler-Sonographie, welche im Verlauf abnimmt und sich schließlich normalisiert. Persistierende Hyperämiezonen im Heilungsverlauf können einen verzögerten Verlauf und z. B. eine Reaktivierung von chronisch-fibrotischem Narbengewebe aufzeigen, woraufhin die Belastung in der Reha angepasst werden sollte und ggf. weitere Maßnahmen wie gezielte Infiltrationsbehandlung – wie unten aufgeführt – durchgeführt werden können ([11]; Abb. 4c).

## Dynamischer Ultraschall

**Figure 5:**
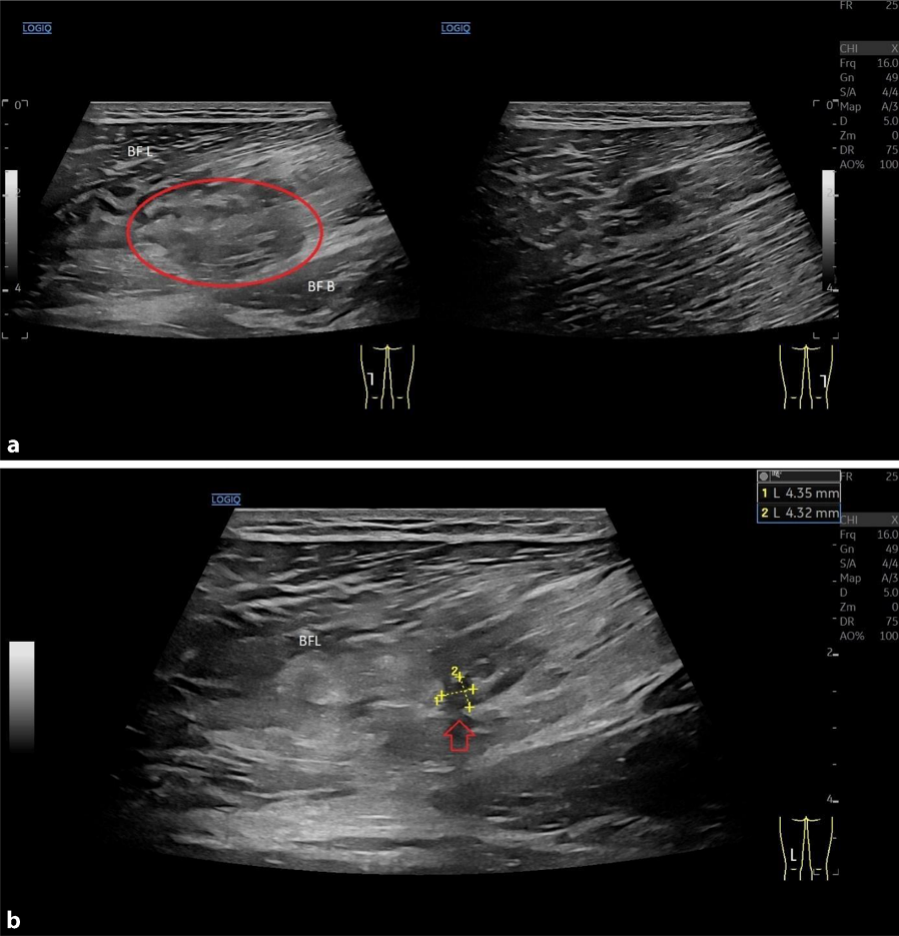

Ein großer Vorteil der Ultraschallbildgebung gegenüber der MRT ist die Möglichkeit eines dynamischen Ultraschalls. Hierbei werden die Muskulatur und das Läsionsareal unter konzentrischer Muskelkontraktion dargestellt. Die Funktion des Narbengewebes (z. B. Adhäsionen und Dysfunktionen) sowie residuale strukturelle Gewebsläsionen können sichtbar gemacht werden, welche im statischen Zustand nicht einsehbar sind. Bei der Erstdiagnostik kann diese Technik ebenfalls zur Detektion von kleinen strukturellen Verletzungen genutzt werden ([10, 11, 35]; Abb. 5a, b).

## Elastographie

**Figure 6:**
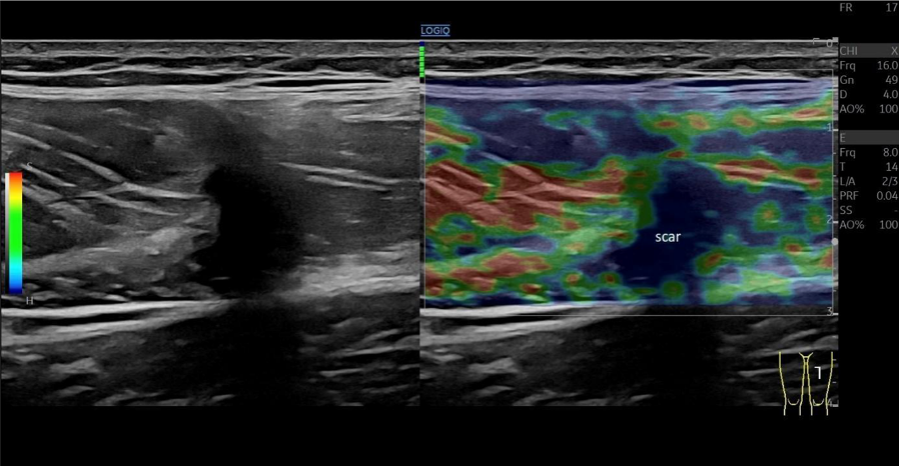

Die Elastographie ermöglicht es, die Elastizität von Gewebe zu messen. Hierbei sind akute Verletzungen als Zonen erhöhter Elastizität sichtbar. Diese können auch kleinere Verletzungen, insbesondere im Vergleich zur Gegenseite, identifizieren, welche in der normalen Bildgebung (B-Mode) nur unzureichend abgrenzbar sind. Im weiteren Verlauf kommt es dann zu hartem, hypoelastischem Narbengewebe, teilweise mit hyperelastischen Rändern (Abb. 6), wobei sich gegen Ende des Heilungsprozesses bei normalem Verlauf ohne exzessive Narbengewebsbildung ein eher heterogenes Elastographiemuster bildet. Die Persistenz von ausgedehntem hypoelastischem Narbengewebe kann auf ein erhöhtes Rezidivrisiko im hyperelastischen Randbereich hinweisen. Die Validität dieser Untersuchungen ist jedoch noch Gegenstand der weiteren Forschung [5].

## MRT-Verlaufskontrollen

Die MRT ist zur Verlaufskontrolle nur bedingt zu empfehlen, da über sehr lange Zeit (mehrere Monate) Signalabnormalitäten bestehen, auch wenn der Spieler schon wieder in die volle Belastung beim Training und Spielbetrieb zurückgekehrt ist [10, 14, 25]. Für die Durchführung von seriellen Untersuchungen eignet sich die MRT ohnehin nur begrenzt. In unserer Praxis wird sie bei unklaren, stagnierenden, nicht durch Ultraschallbildgebung erklärbaren Verzögerungen und persistierenden Schmerzen sporadisch eingesetzt.

Es bleibt zu bemerken, dass weder die MRT noch der Ultraschall eine zweifelsfreie Aussage über eine sichere Rückkehr zur vollen sportlichen Belastung treffen können. Hierbei spielen eine Vielzahl von Variablen eine Rolle, welche nicht durch eine isolierte Bildgebung erfasste werden können (z. B. Fitness, Kraft im Seitenvergleich, Alter, Spielertyp, mentale Fähigkeiten, Berater, Verein, mögliche systemische Erkrankungen, Substratmangel) [11, 35].

## Komplikationen bei Muskelverletzungen

Serielle Ultraschalluntersuchungen bieten die Möglichkeit zur frühzeitigen Erfassung therapiebedürftiger Komplikationen.

### Myositis ossificans

**Figure 7:**
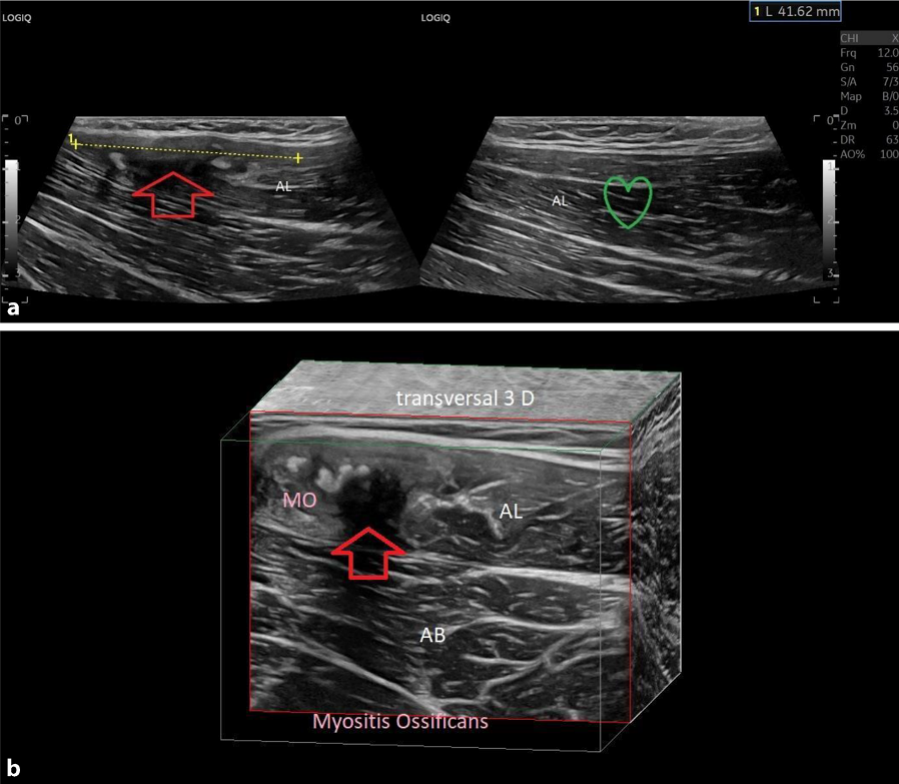

Diese tritt vorwiegend bei großen Hämatomen vor allem in Knochennähe wie z. B. einer direkten Muskelverletzung (Prellungen) des Vastus intermedius oder auch im proximalen Oberschenkel z. B. der Adduktoren oder der proximalen Quadrizepsmuskulatur auf. Der Ultraschall bietet die Möglichkeit einer Frühdetektion nach ca. 1–2 Wochen (Abb. 7a, b) mit hyperechogenen Zonen teilweise mit „quellenferner Schallauslöschung“, respektive Schallwellenreflexion und signifikanter entzündlicher Aktivität im Läsionsareal. Im Röntgen können diese erst nach ca. 6 Wochen erfasst werden und in der MRT zeigen sich in dieser Zeit nur unspezifische heterogene Veränderungen [4, 14, 32]. Entscheidend ist die Früherkennung und umgehende Therapie z. B. mit NSAR und ggf. Infiltrationen mit Traumeel®, Sanuvis® oder PRP („platelet rich plasma“) und dem Verbot für jegliche tiefe Gewebemassagen durch Therapeuten. Ein allfälliges Hämatom sollte ebenfalls umgehend punktiert werden. Auch wenn die Datenlage für dies Maßnahmen begrenzt ist, so kann aus der Erfahrung gerade bei Erfassung von sehr frühen Stadien durch rechtzeitiges Eingreifen ein Fortschreiten verhindert oder sogar die Verkalkungs- und Ossifikationsherde teilweise wieder aufgelöst werden. Im Falle einer ausgeprägten Myositis ossificans droht dem Sportler teilweise ein Ausfall bis zu 6 Monaten [4].

### Hämatome

Hämatome können sonographisch gut dargestellt und therapiert werden. Es wird empfohlen, Hämatome über 3–5 ml ultraschallnavigiert direkt zu aspirieren [11]. Diese könnten zwar im Verlauf des Heilungsprozesses wieder abgebaut werden, allerdings besteht die Gefahr zur Bildung von fibrotischem starrem Narbengewebe, welches dann persistierende funktionelle Störungen nach Abheilung der Verletzung verursachen kann. Eine ultraschallnavigierte Punktion ist problemlos und sicher durchführbar. Ein abwartendes Verhalten empfiehlt sich nicht, da sich das Hämatom kurzfristig organisieren kann und dann nicht mehr punktionsfähig ist [35]. Aufgrund des Rezidivrisikos sollte nach 5–7 Tagen eine Erfolgskontrolle erfolgen und die Therapie bei Bedarf wiederholt werden. Postinterventionell empfiehlt sich grundsätzlich eine Kompressionstherapie für 1–2 Tage z. B. mittels eines Zinkleimverbandes und elastischen Bandagen. Gleiches Prozedere gilt für Hämatoserome und Serome. Bei hartnäckigen Rezidiven, insbesondere bei intermuskulären Hämatomen, z. B. nach Gastroknemiusverletzungen, kann eine kleine Menge eines Steroidpräparats nach Aspiration appliziert werden, um schwere Dysfunktionen der betroffenen Muskelgruppen zu verhindern [12].

### Posttraumatische intramuskuläre Thrombosen

**Figure 8:**
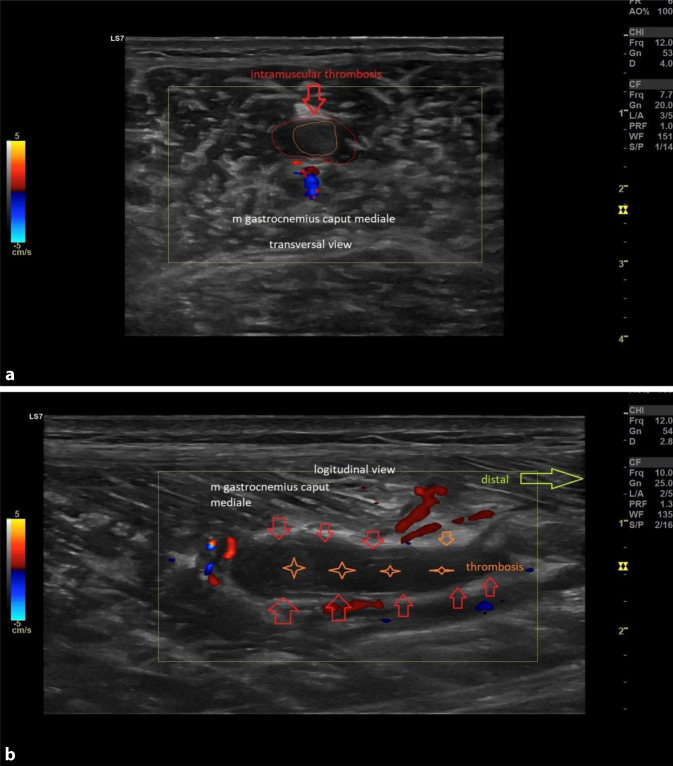

Diese sind ebenfalls mittels Ultraschalls (Zwei-Punkt-Kompressionsultraschall und Doppler-Sonographie) gut darstellbar und bedürfen einer umgehenden Therapie (Abb. 8a, b). Klinisch klagt der Sportler oft nach einem initial guten Verlauf über neue Schmerzen, möglicherweise nicht direkt über dem Läsionsareal. Auch direkte Muskelkontusionen oder repetitive Traumata im Unterschenkelbereich können über vasospastische Effekte eine Venenthrombose auslösen. Häufig treten diese nach Verletzungen des medialen M. gastrocnemius auf [30]. Aufgrund der schwierigen Therapie im Hinblick auf eine Orale-Antikoagulation-Behandlung empfiehlt sich eine enge Zusammenarbeit mit spezialisierten Sportangiologen, da oftmals eine Standardtherapie nicht mit dem Fußballsport als Kontaktsportart vereinbar ist.

Weitere Komplikationen sind Muskelhernien, nicht funktionelles fibrotisches Narbengewebe und Kompartmentsyndrome. Diese können ebenfalls im Ultraschall erfasst und entsprechend therapiert werden [6].

## Additive Injektionstherapien

Generell empfiehlt es sich, jegliche Interventionen unter Ultraschallnavigation durchzuführen. Hierbei können die Therapien punktgenau unter Vermeidung von Verletzungen von Gefäßen, Nerven und intakten anatomischen Strukturen (z. B. Sehnen, angrenzendem gesundem Gewebe und Muskulatur) durchgeführt werden. Bei sachgerechter, steriler und „No-touch“-Technik ist das Komplikations- und Infektionsrisiko verschwindend gering [6, 18].

### „Platelet rich plasma“ (PRP)

Die positive Wirkung von PRP wurde für viele Indikationen bereits durch zahlreiche Studien belegt. Hierbei konnten zahlreiche positive Effekte, wie Schmerzreduktion, Normalisierung einer allfälligen überschießenden entzündlichen Aktivität und Beschleunigung regenerativer Effekte, beobachtet werden. Bei der Herstellung, Zusammensetzung und Applikation des Plasmas herrscht jedoch große Heterogenität, sodass es insbesondere bei Verwendung von Muskel- und Sehnenverletzungen kein Konsens gibt. Dennoch kommt PRP in der Praxis v. a. bei höhergradigen Muskelverletzungen speziell bei Mitbeteiligung von Sehnenstrukturen häufig zum Einsatz [2, 16, 29]. Bei Verwendung eines leukozytenarmen Plasmaproduktes wurde auch bei häufiger Anwendung in der Praxis noch kein Effekt einer überschießenden Fibrose beobachtet. Aus der Erfahrung heraus besteht die Möglichkeit der Anwendung bei allen strukturellen Muskelverletzungen. Je nach Schweregrad der Verletzung wird dies 3‑ bis 5‑mal in den ersten 2 Wochen ultraschallnavigiert intraläsional appliziert. Auch bei Rezidivverletzungen hat sich die Anwendung in der Praxis bewährt. Speziell bei Ausbildung eines insuffizienten Narbengewebes mit entsprechenden Re-Rupturen („soft scar tissue“) kann der Einsatz aus eigener Erfahrung empfohlen werden. Postinterventionell erfolgt wie bei den meisten Interventionen eine Kompressionstherapie für 1–2 Tage. Schwere Nebenwirkungen oder andere negative Effekte wurden bei zahlreicher Anwendung nicht beobachtet. Der Einsatz bleibt jedoch jedem Therapeuten in Diskussion und der Präferenz des Sportlers und seinem Team selbst überlassen.

### Traumeel® und Sanuvis® (Acidum L(+)-lacticum)

Traumeel® ist eine Kombination aus verdünnten Pflanzen- und Mineralienextrakten und hat bioregulatorische Eigenschaften wie mögliche beschleunigte Wundheilung, Reduktion von Ödemen durch Reduktion von mikrovaskulären Leckagen und Regulation überschießender entzündlicher Prozesse von Gewebestrukturen [27]. Auch hier ist die Studienlage limitiert, es kann jedoch bei Muskel- und Sehnenverletzungen analog zu PRP zum Einsatz kommen. Eine Kombination mit anderen Stoffen wie z. B. PRP und Sanuvis® ist ebenfalls möglich. Schwere Nebenwirkungen treten bei der Anwendung in der Regel nicht auf. Allfällige lokale Hautreaktionen (Rötung, Juckreiz oder Hitzegefühl) sind meist selbstlimitierend [28].

Sanuvis® werden ebenfalls antientzündliche und analgetische Eigenschaften in der Regulation des pathologischen Milchsäuremetabolismus der rechtsdrehenden Milchsäure (D-) zugeschrieben, welche bei erhöhtem Energiebedarf der Zelle entsteht [33]. Der Einsatz in der täglichen Praxis hat sich trotz fehlender systematischer Studien bei beiden Präparaten sowohl alleine als auch in Kombination mit anderen regenerativen Injektionen in der Praxis bewährt.

### Muskelrelaxanzien (z. B. Pridinol)

Muskelrelaxanzien können bei hartnäckigen posttraumatischen Myogelosen zum Einsatz kommen. Diese sollen zur Reduktion der Spannung proximal und distal des Läsionsareals führen [9]. Ob sich hierdurch eine verbesserte Heilung oder Regeneration erzielen lässt, ist nur schwer objektivierbar. Trotz fehlender Studienlage kommt es in der Praxis bei entsprechender Indikation zum Einsatz.

### Lokalanästhetika (LA)

Die Applikation von LA soll durch Blockade von Nervenaxonen den Schmerz reduzieren und so zu einer Detonisation der Muskulatur führen. Dieser Effekt wird jedoch durch die Nebenwirkungen von LA auf die Muskulatur aufgrund ihrer myotoxischen Eigenschaften mit Kurz- und Langzeitschäden und Myonekrosen bis über 4 Wochen nach Applikation überschattet, weshalb wir empfehlen, diese nach Möglichkeit bei Muskelverletzungen nicht einzusetzen [24].

## Neue Therapieoptionen mittels Stoßwellen

Zusätzlich zu klassischen Behandlungsmethoden des physiotherapeutischen Spektrums kann eine Stoßwellenbehandlung erfolgen. Bei niedriggradigen Verletzungen (≤ Grad 3A) und therapieresistenten chronifizierten Muskelbündelverletzungen (Grad 3B) konnte ein positiver Effekt auf einen schnelleren „return to play“, eine Schmerzreduktion und eine Verkleinerung von Hämatomen beschrieben werden. Zusätzlich zeigte sich ein positiver Effekt mit Reduktion der Rezidivrate bei niedriggradigen Verletzungen (bis maximal Muskelfaserriss; Grad 1–3A). Hierbei können sowohl radiäre als auch fokale Stoßwellen zum Einsatz kommen [15].

## Fazit für die Praxis

- Muskelverletzungen bedürfen zur Verifizierung einer Bildgebung.
- Sowohl die MRT als auch der Ultraschall haben hierbei ihren Stellenwert.
- Bei richtigem Einsatz eines modernen hochauflösenden Ultraschalls kann bis auf wenige Ausnahmen auch im Profibereich auf eine MRT-Bildgebung weitestgehend verzichtet werden.
- Ein großer Vorteil des Ultraschalls ist die Möglichkeit einer dynamischen Untersuchung. Auch die Doppler-Sonographie und die Elastographie unterstützen die Ultraschallbildgebung mit wertvollen Zusatzinformationen.
- Sonographische Verlaufskontrollen bieten sowohl die Möglichkeit der Monitorisierung des Heilungsprozesses als auch des frühzeitigen Erkennens von Komplikationen, mit der Option diese umgehend zu behandeln.
- Bei unklaren Befunden, tiefen Strukturen oder Verletzungen des M. soleus bleibt die MRT weiterhin die Bildgebung der Wahl.

## Abkürzungen

BAMIC: British Athletics muscle injury classification
BSC YB: Berner Sport Club Young Boys
LA: Lokalanästhetika
NSAR: Nichtsteroidale Antirheumatika
PRP: „Platelet rich plasma“
US: Ultraschall
